# Lived experiences of South African rehabilitation practitioners during coronavirus disease 2019

**DOI:** 10.4102/ajod.v13i0.1229

**Published:** 2024-01-12

**Authors:** Sadna Balton, Mershen Pillay, Rizqa Armien, Annika L. Vallabhjee, Elani Muller, Mark J. Heywood, Jeannie van der Linde

**Affiliations:** 1 Department of Speech Therapy and Audiology, Chris Hani Baragwanath Academic Hospital, Soweto, South Africa; 2 Department of Speech, Language Pathology, Faculty of Health Sciences, University of KwaZulu-Natal, Westville, South Africa; 3 Department of Speech, Language Therapy, Institute of Education, Massey University, Auckland, New Zealand; 4 Department of Occupational Therapy, Symphony Way Community Day Centre, Cape Town, South Africa; 5 Effective Care Research Unit, East London, South Africa; 6 Nelson Mandela School of Public Governance, University of Cape Town, Cape Town, South Africa; 7 Department of Speech-Language Pathology and Audiology, Faculty of Health Sciences, University of Pretoria, Pretoria, South Africa

**Keywords:** COVID-19, lived experiences, rehabilitation practitioners, mental health, innovation, leadership South Africa

## Abstract

**Background:**

In South Africa, the sharp rise in people with severe illness because of coronavirus disease 2019 (COVID-19) in early 2020, meant that health systems needed to adapt services and operations, including rehabilitation services. Important insights into the lived experiences of rehabilitation personnel enacting these adaptations in an African context are limited.

**Objectives:**

The aim of this study was to explore the lived experiences of rehabilitation practitioners working in the public sector in South Africa during the COVID-19 pandemic.

**Method:**

A phenomenological approach and a duo-ethnographic design were used. A recruitment letter was circulated requesting volunteers. Maximum variation sampling was used to select the 12 participants of this study. Data were collected through interviews via Zoom, and critical conversations were facilitated by a non-rehabilitation partner who is known for challenging health inequities. The interviews were audio-recorded and transcribed verbatim. Data were analysed through elements of qualitative content and thematic analysis. Data were coded, categorised, clustered into concepts and formulated into themes.

**Results:**

Three themes were identified: (1) ‘Management became the enemy’, (2) ‘Tired of being resilient’ and (3) ‘Think out of the box…think on our feet’.

**Conclusion:**

The results of this study highlighted new ways of practice, innovative adaptations, and usage of resources and platforms.

**Contribution:**

This study highlights the re-imagining of accessible rehabilitation services that could lead to deeper onto-epistemological shifts amongst the rehabilitation practitioners.

## Introduction

In December 2019, people presented to healthcare facilities in Wuhan, China, with severe pneumonia of unknown aetiology (Lew, Oh-Park & Cifu 2020). As the influx of patients continued and investigations were done, the coronavirus disease 19 (COVID-19), which was caused by severe acute respiratory syndrome coronavirus 2 (SARS-CoV-2), was identified (De Biase et al. 2020; Lew et al. 2020). Globally, the number of cases and deaths was climbing steeply because of COVID-19 (Ngeh et al. 2020). The president of South Africa announced a nationwide lockdown; social distancing principles and mask-wearing became mandatory (Nkonki & Fonn 2020).

The sharp rise in severely ill patients meant that health systems across the world needed to adapt services and normal operations to cater for the emerging health needs of communities (De Biase et al. 2020). This led to many challenges that healthcare workers had to face in healthcare institutions. Challenges included a shortage of beds, ventilators, human resources, and the limited availability of personal protective equipment (PPE) and other medical equipment (Hill et al. 2020; Lew et al. 2020). Certain health, rehabilitation and social services came to a halt as resources had to be redistributed to assist with the treatment of COVID-19 patients (Lew et al. 2020). Because of a lack of close collaboration between sectors at all the different levels of management, the identification and agreement on what is deemed priority essential services, resulted in a breakdown in the continuity of care (Modisenyane et al. 2021).

Many rehabilitation practitioners were required to function outside their scope of practice to assist with the burden of the pandemic; this included assisting with the screening and triaging of patients for COVID-19 (Adams et al. 2021). Rehabilitation practitioners also had to work in dedicated COVID-19 units, where they had to act quickly with limited protocols and guidelines available to inform the acute and lifesaving medical care provided to COVID-19 patients (Amatya & Khan 2020b; De Biase et al. 2020; Lew et al. 2020). In some COVID-19 units, physiotherapists were responsible for providing interventions targeting cardio-respiratory, pain and musculoskeletal dysfunction, while speech therapists were providing dysphagia-related interventions (Adams et al. 2021; Hassem et al. 2022). Occupational therapists offered mental health services to patients, as the demand for mental health care escalated because of pandemic-induced stress and anxiety around the pandemic (Firshman, Hoffman & Rapolthy-Beck 2020). At a later stage, research and guiding documents from professional bodies or associations assisted with planning and refining service delivery during the pandemic (Malec et al. 2020; Ngeh et al. 2020; Sheehy 2020).

The emerging needs of patients with and affected by COVID-19 were multifold: patients with severe illness, in need of ventilation for prolonged periods of time experienced cognitive impairment at the time of discharge with functional deficits for up to one year (Lew et al. 2020). Increasing concerns such as major depressive disorder, anxiety and post-traumatic stress disorder (PTSD) were also noted among some patients directly affected by the disease (De Biase et al. 2020; Lew et al. 2020; Lund et al. 2020). Furthermore, a significant number of patients had word-finding difficulties, voice changes and difficulties with swallowing (Royal College for Speech, Language Therapy [RCSLT] 2022). The cessation of rehabilitation services caused further disease leading to increased impairment, poverty, social exclusion and poorer functional outcomes (Lund et al. 2020; Ngeh et al. 2020). This correlation is unsurprising, given the well-established bidirectional relationship between disability and poverty (Pinilla-Roncancio 2015). Decreased access to healthcare services globally (World Health Organization [WHO] 2021) led to rehabilitation practitioners finding innovative ways to provide therapy to their patients despite the COVID-19 restrictions. Telerehabilitation was identified as an existing method that could bridge the gap between patients and their access to rehabilitation services (Doraiswamy et al. 2020; Pillay et al. 2020). Despite the benefit of telerehabilitation during the COVID-19 pandemic, challenges included limited access to technology, high cost of data and a lack of implementation guidelines on telepractice in public health care; this influenced the effectiveness of these novel approaches (Leochico, Rey-Matias & Rey-Matias et al. 2022; Pillay et al. 2020).

While some countries have described their health system changes and ways of providing rehabilitative services to their patients, important insights into how rehabilitation personnel executed these changes in an African context remain limited (Ngeh et al. 2020). A number of studies have been conducted since the outbreak of the COVID-19 pandemic, the majority of which explored the impact of the virus on, but not limited to, service delivery (Landes et al. 2020; Lebrasseur et al. 2021; Patterson et al. 2020), the manifestation of the virus itself (Ahmed et al. 2020; Kamal et al. 2021; WHO 2020), and its impact on the mental health of health care workers (Cag et al. 2021; Fiejit et al. 2020; Robertson et al. 2020; Temsah et al. 2020). Jow et al. (2022) conducted a cross-sectional study looking at the mental health of physiotherapists, speech therapists and occupational therapists, and found a high impact of COVID-19 on the occupational stress and psychological well-being of the participants. A global survey conducted by Cag et al. (2021) found that anxiety scores significantly increased in relation to younger female health workers (Ned et al. 2020; Pillay et al. 2020), which constitute most rehabilitation professionals in South Africa.

Literature review showed a focus on the lived experiences and viewpoints of nurses (Fathi et al. 2020; Liu et al. 2020; Iheduru-Anderson 2020; Karimi et al. 2020; Roberts, Knestrick & Resick 2021), doctors (Fathi 2020; Liu et al. 2020; Yarrow & Pagan 2020) and patients (Aliyu et al. 2021; Portocolone 2021). Yet, limited research describes the lived experiences of rehabilitation practitioners. Understanding their lived experiences could improve our understanding of what it means to provide rehabilitation services under these conditions (Dierckx de Casterle et al. 2011). This requires critical reflexivity which accounts for the interconnectedness between the personal, interpersonal, methodological and contextual factors for both the participants and researchers (Francisco et al. 2023; Gilgun & Jane 2008). This reflexivity is contextualised within the spirit of ubuntu which asserts that ‘to be a human being is to affirm one’s humanity by recognising the humanity of others’ (Ramose 2002:37).

The aim of the study was to explore the lived experiences of rehabilitation practitioners working in the public sector in South Africa during the COVID-19 pandemic.

## Research methods and design

A phenomenological approach using critical conversations rendered an opportunity for capturing the lived experience of participants (Frechette et al. 2020). Phenomenological research ‘is a return to embodied, experiential meaning, to seek fresh, complex, vivid descriptions of a “phenomenon” (a human experience in all its complexity) as it is concretely lived’ (Finlay 2009). These lived experiences bring together the stories of people to enrich the understanding and scope of the phenomenon (Honey et al. 2020). Diverse manifestations across African, Asian, Latin American, Caribbean and other contexts emphasise oral history. The concept of ‘the story’ has now evolved into a recognised ‘narrative turn’ in research, a development that has been both welcomed and critiqued within disciplines such as medicine, nursing, rehabilitation and many other health science disciplines (Giraldo-Pedroza et al. 2021; Kathard et al. 2004), potentially because of its divergence from quantitative research. Our study design was supported by the mapping of personal experiences using a method developed from a study on communication sciences called ‘critical conversations’ (Pillay 2003). The goal of a critical conversation is to overtly foreground issues of social, cultural and political gravitas when producing and interpreting data. Critical conversations harmonise with this narrative-biographical design as it relies on dialogue to represent experience to reconceptualise and regenerate the meaning. These are also essentially reflexive processes that apply to critical conversations and shift across technical/descriptive, interpretive approaches (Sveningsson & Karreman 2021).

This study investigated the experiences of physiotherapists, occupational therapists, speech and language therapists, and audiologists in the public sector across South Africa. These practitioners’ work in diverse settings across the provinces and between urban or rural contexts. These contextual variations have resulted in disparities in resources such as human, financial and physical. These resource inequities have historical roots tied to the allocation of resources for different racial groups in South Africa. This legacy, in accordance with separate development and its political overflow into healthcare, resulted in unequal healthcare services for people of colour, often provided by under-resourced health care facilities (De Villiers 2021). In contrast, relatively well-resourced facilities were geared towards serving the minority of white people in South Africa, echoing historical biases.

### Sampling method

Maximum variation sampling (Given 2008) was used to select a wide diverse study sample. In this study, participants were purposefully and maximally different from each other by race, experience, job title (e.g. manager, clinical practitioner) and location or site (e.g. province). Race and gender were considered because these are significant social and political identities that interact with professional practices (Abrahams et al. 2019).

### Ethical considerations

Prior to recruitment, a human research ethics committee clearance was obtained from the review board of the University of Cape Town (REF:569/2021). A recruitment letter was circulated to the National Public Sector Rehabilitation Forum to all occupational therapists, speech-language therapists, physiotherapists and audiologists, describing the study and asking volunteers to contact the principal investigator. Eligibility was determined by the research team, consent was obtained, and participants were enrolled in the study.

### Inclusion criteria

The inclusion criteria for rehabilitation practitioners were those: (1) whose primary practice site is in the public sector in clinical and/or managerial (service) positions; (2) who have practised with/for COVID-19 patients; and (3) who self-declared their willingness and ability to participate in a critical conversation.

### Study population and sampling strategy

The study sample included 12 participants with 4 being in management positions. The professional breakdown included four occupational therapists, three physiotherapists, three speech-language therapists and two audiologists. Of these, three were male and nine were female. Four participants were from Gauteng, three from Mpumalanga, two from the Western Cape, two from Kwa-Zulu Natal, and one from North West province. Three rehabilitation practitioners had 5 and less years of experience, two had between 6 and 10 years and four had between 16 and 20 years of experience.

### Data collection

Twelve individual critical conversations were conducted which took an average of an hour each. At the time of each critical conversation, participants received both oral and written information about the study, including their right to withdraw their participation at any point without consequences. Written informed consent was obtained from all the participants. The conversations were conducted via Zoom by an individual who was not a member of the rehabilitation professions, and who is a natural disruptor, which is required to facilitate critical conversations and to develop an emic-etic balance in the methodological processes typical of critical conversations (Lambert, Glacken & McCarron 2011; Macnamara 2021; Olive 2014). Emic and etic perspectives are important in ethnographic research so that it captures the interaction between the interviewer and participants to explore multiple perspectives (Macnamara 2021; Olive 2014).

Twelve participants were asked to share their experiences, based on their unique perspectives and, as these were critical conversations, specific, structured questions were not predetermined. The questions differed based on the information obtained during each conversation which ranged from 50 to 60 min. A short introduction was provided by the lead conversation partner to situate the study and to position their role as a health activist and journalist who was not trained in any of the rehabilitation professions. At the end of the conversation, participants were provided the opportunity to correct misunderstandings or add additional information. Conversations were audio-recorded using Zoom online meeting software, and transcribed verbatim using Otter.ai, a speech-to-text transcription software that uses artificial intelligence (AI). Zoom allows for the safe protection of data for research because of its ability to securely record and store sessions for the purpose of collaboration (Archibald et al. 2019). Transcriptions were then rechecked for syntactic and content accuracy by the researchers shortly after each critical conversation. Interviewee transcript review (Hagens, Dobrow & Chafe et al. 2009) was utilised where participants were provided with their verbatim transcripts to verify for accuracy and correct any errors. The audio recordings were stored in a password-protected file on the lead researchers’ computer and managed in accordance with University of Cape Town’s data management policy. Each participant’s audio-recording and transcript were de-identified and labelled with a code (numerical) to maintain confidentiality, and only the primary investigator had knowledge of the participants’ code.

### Data analysis

Data were analysed after all critical conversations were completed, and a thematic analysis was conducted using steps described by Braun and Clark (2019). All data obtained were initially read to provide a holistic view of the reported lived experience. Data were coded (substantive and axial coding) resulting in an extensive list of codes; the codes were categorised, clustered into concepts, and finally formulated into three themes. Researchers then subsequently discussed, critiqued, collaborated and concluded on any divergent opinions concerning the categorisation and themes.

This reflection allowed the researchers to evaluate their subjectivity, belief systems and underlying biases which contributed to the overall trustworthiness of the study (Delve & Limpaecher 2022; Olmos-Vega et al. 2023).

## Results and discussion

Exploring the lived experiences of rehabilitation practitioners allowed for data to be generated from the field towards specific themes or theoretical perspectives of rehabilitation practice in South Africa during the COVID-19 pandemic. Three themes were identified through the content and thematic analysis: (1) ‘Management became the enemy’, (2) ‘Tired of being resilient’, and (3) ‘Think out of the box…think on our feet’.

### ‘Management became the enemy’

The fragility of healthcare systems and its leadership was exposed by the COVID-19 pandemic, which included the lack of leadership, limited guidance and poor communication from top management. While there is no playbook for leadership in a pandemic, positive and collective leadership that is ‘authentic, aware, adaptive, flexible, as well as trusted, engaged and compassionate’ is crucial (Anjara et al. 2021; Hill et al. 2020). Collective leadership is displayed by joint participation in decision-making by all team members who complete tasks that were reserved for a hierarchical leader (Anjara et al. 2021; Edwards & Bolden 2023).

In this study, top management’s response was described as a ‘fragmented, reactive response to COVID-19’. The anger of rehabilitation practitioners is exemplified in the responses of Participants 5 and 6:

‘They haven’t experienced it. It’s nice for people, for people to sit in the offices and tell us what to do. But if you are not working alongside me, and you’re not seeing what I’m seeing, you can’t tell me in terms of how to do things better.’ (P5, Male, Physiotherapist)

And so, management also became the enemy if I can call it that, at some point. “You guys are making the decision for us. You’re not considering our point of view. You’re not going and working in the actual wards; you are not in the frontline. You’re sitting behind your desk and your computers in the office in the safest space.’

‘But some of them just still wanted to be I’m the manager, I’m the boss, you just need to do x, y, and z.’ (P6, Male, Audiologist)

Mather (2020) states that leaders need to act with urgency, take responsibility by responding to mistakes, adapt and constantly update, communicate with clarity and transparency, and think outside silos. Managers who were interviewed highlighted these behaviours as they reported that they led their teams with the sense of urgency that was required. They achieved this by initiating collaboration with other health care workers in the wards and by developing systems within their departments to address workload and well-being challenges. Transparency and communication were the key strategies utilised by managers. Other strategies included daily meetings to touch base and share any new information received, developing protocols, establishing clear and accessible communication channels, and responding from a point of ‘humanity’. This example of leadership exemplifies some of the characteristics as described by Mather (2020) earlier:

‘I think one of the things of being a manager, you don’t have that option to run away. So, I felt like, I have to experience it, so I know how it feels so when I give it to somebody else, I will tell them this is what to expect, this is how it feels like, be aware of certain things.’ (P12, Female, Manager)

One of the managers stated that there was a lot of resentment directed towards them; however, they did not receive much guidance from their hospital management or province as validated by a provincial manager in the quotation below:

‘We did not provide sufficient support and sufficient guidance in terms of what needed to happen during that phase to ensure that people are supposed to do the same thing across the province.’ (P1, Male, Manager)

The lack of collective leadership and barriers to service delivery led to ‘inverted decision-making’ by rehabilitation practitioners; that is, the traditional hierarchical approach to decision-making was flipped. Collective leadership refers to an approach that allows us to understand power relations within the rehabilitation context, as discussed below by a variety of practitioners such as clinical leaders or their managers towards transforming healthcare services in the future. Frontline rehabilitation practitioners initiated collaborative team decision-making to find solutions to service delivery challenges, thereby deviating from ‘the traditional command and control’ leadership style (Anjara et al. 2021:2).

**FIGURE 1:**
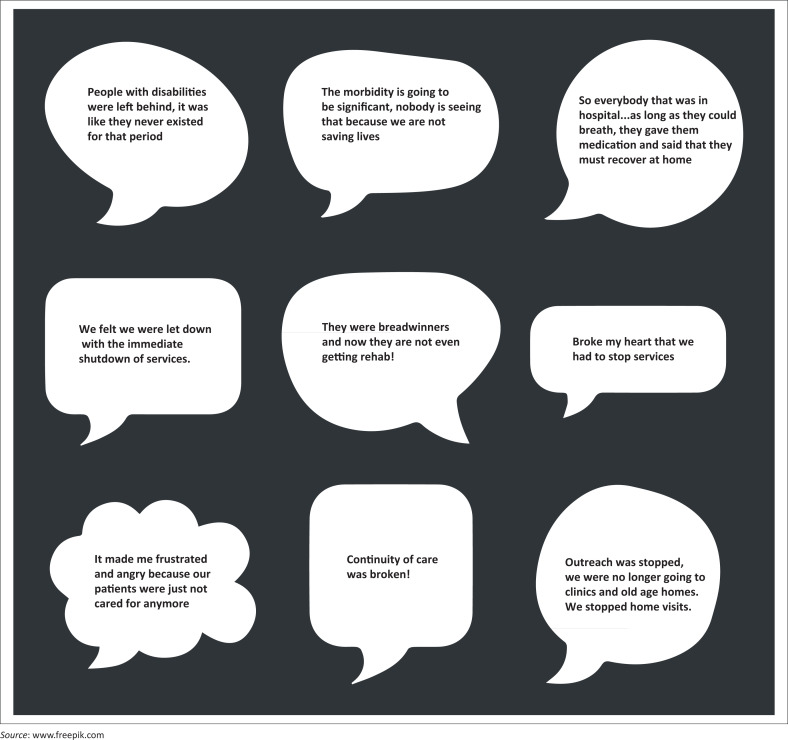
Participant’s responses on rehabilitation services being stopped.

Work model rotations, the practice of alternating schedules, shifts and working hours, were instituted within some rehabilitation teams, and were later adopted by management using a consultative decision-making model. This response was used so that rehabilitation practitioners could ensure that service delivery continued, despite changing and uncertain circumstances. ‘Risk rating with a heart’ was implemented to safeguard team members with comorbidities. This implies that a decision-making process was used that not only considered objective risk assessments but also considered the well-being and needs of the individual involved. This model was unfortunately not carried over at all levels of service delivery, highlighting the siloed response in leadership. Rehabilitation practitioners felt pressured to continue providing services to all citizens at all levels of healthcare as the struggling healthcare system was crippled further by the COVID-19 pandemic (McKinney, McKinney & Swartz 2021; Uys et al. 2021). While there was an international call for rehabilitation to be included in disaster management planning (Amatya & Khan 2020b), rehabilitation practitioners across disciplines in this study reported that rehabilitation services were ‘at the bottom of the food chain’. The statements in Figure 1 highlight the emotions and frustrations felt because of rehabilitation outpatient services being stopped, and inpatients being prematurely discharged because of COVID-19.

While the directive was to stop services, there was a commitment to finding alternate ways of reaching patients (Ilyas et al. 2021). Participants also expressed their concern about the socioeconomic impact of this exclusion and its disproportionate effects on people with disabilities living in poverty (Banks et al. 2021). This concern was highlighted by Participant 10:

‘“You know, people with disabilities have high levels of comorbidities, higher levels of poverty, higher levels of risk of developing secondary complications”… “So, we started fundraising for food parcels. We worked with a local faith-based organisation. Just a few food parcels, and then we distributed them”.’ (P10, Female, Physiotherapist)

Many participants commented that they experienced an identity crisis related to their role within the healthcare system during COVID-19. There was a general sense of underappreciation of the role of rehabilitation as part of the health care systems response to COVID-19. Participants expressed that their voices were not heard and that they were often not consulted when decisions were made:

‘I think we had a little bit of an identity crisis in terms of being essential, where do we fit in this crisis? You know, we don’t deal with these life and death things.’ (P2, Female, Occupational Therapist)

‘…very often we are not even afforded that platform to say for you, what do you think will happen to ensure that the people that you serve still receive the service, so very often that doesn’t come through, so we are often forgotten in this thing.’ (P1, Male, Manager)

Participants further highlighted a sense of being forgotten, when they shared examples of their difficulty trying to access PPE. Participant 8 expressed their disappointment when describing their experience during the first wave:

‘We would come to work in the morning, and you have to sign a register to get a mask. And that mask has to be used until physically, it was spoiled, or you couldn’t use it again.’ (P8)

A study conducted at a tertiary hospital in South Africa also found that health care workers reported psychological distress related to the perceived barriers to infection control practices (Lee et al. 2022). This feeling of being undervalued and underappreciated had a negative impact on the well-being of many rehabilitation practitioners.

### ‘Tired of being resilient’

Rehabilitation practitioners, like all other health workers, were expected to ‘step up, be brave, and provide care and comfort’ (Iheduru-Anderson 2020) to those affected by COVID-19 while faced with the challenge of managing their personal/human response to the pandemic.

Participants in this study stated that the critical conversations provided a debriefing session for them. Participant 2 summarised the sentiment of most participants when stating that:

‘I was just excited about this interview in general, because I just feel like it’s a platform to be heard by people who understand and who would understand rehabs perspective and rehabs background, and not just look at it in terms of you a health care worker and that’s really bad.’ (P2, Female, Manager)

Like Chersich et al. (2020) and Dawood, Tomita and Ramlall (2022), we also noted participants reporting difficulties coping with stress, anxiety and the psycho-emotional impact that the pandemic has had on them.

**FIGURE 2:**
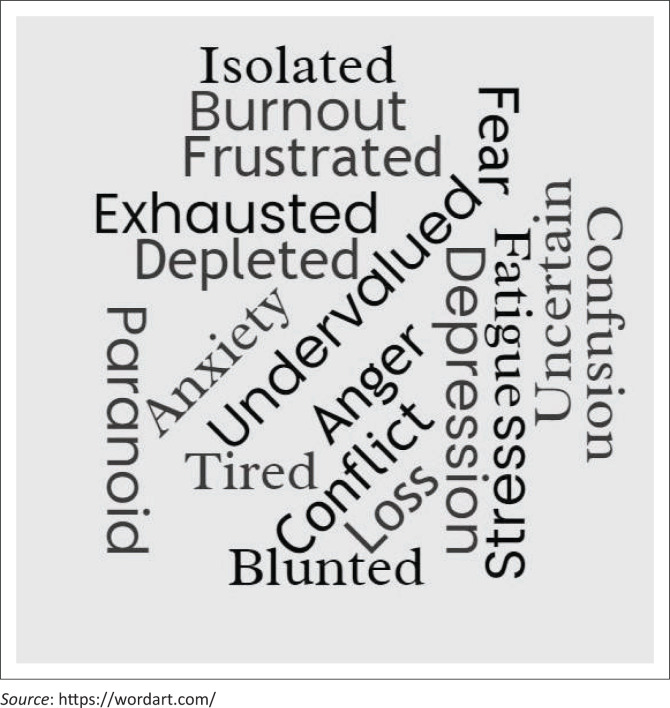
Word cloud of human and personal responses on the coronavirus disease 2019 pandemic.

In Figure 2, participants’ expressed responses across the spectrum of emotions referencing paranoia, feelings of anxiety, anger, frustration, exhaustion, stress, and burnout. Some mentioned developing a blunted affect alongside COVID fatigue, expressing that they were ‘tired of being resilient’ which concur with findings in a study conducted by Jow et al. (2022). Participants stated that their feeling of anxiety was initially related to the lack of PPE, dealing with the uncertainty of COVID-19 on individuals, and its impact on the profession.

Rehabilitation practitioners enacted personal or human responses across their professional and home lives, as evidenced by three significant issues: management of work and family responsibilities, protecting families against possible exposure, and having to make the rules about the safety of family members within the home context. Rehabilitation practitioners, who are predominantly female, reported the added burden of having to continue with their responsibilities at home in addition to online learning for their children. Saragih et al. (2021) found that women healthcare workers faced an increased caregiving burden during the pandemic which impacted their health and well-being. This is highlighted by Participant 7:

‘… I understand my commitments, as a wife, a mother, a daughter, whatever are the figures that you’re playing, and how is it that you’re also able to provide for you, like, provide that aspect of what needs to happen, and try and put those together and get that work life balance. … I feel like work-life balance for women is really, really difficult in the public health sector.’ (P7, Female, Speech therapist and Audiologist)

The fear of infecting family members at home increased the fear and anxiety experienced by participants at work. One participant mentioned that they avoided any contact with their family after they had contracted COVID-19. The lack of understanding at community level further isolated rehabilitation practitioners from visiting family. The added responsibility of keeping families safe and making rules within families to ensure that everyone stayed safe was an additional burden on rehabilitation practitioners. The line between home and work became blurry as COVID-19 was not just something being experienced by the patients they treated at work but was something that affected their families.

The impact of people’s loss was not limited to work, as many had to deal with loss of family members and return to work and continue to function within a highly stressful and demanding setting. This was expressed by Participant 1:

‘It was very stressful, a lot of friends, colleagues, and family members that we buried. I’ve lost two sisters, so it was very sad, but personally I think I’ve just accepted that it’s one of those circumstances and then we just have to accept and move on.’ (P1, Male, Manager)

Undoubtedly the pandemic was scary, an event guaranteed to evoke a range of emotions, particularly fear and anxiety as displayed by the participants responses:

‘A lot of uncertainty, a lot of fear. And fear mongering a lot of my family worrying about me.’ (P5, Male, Physiotherapist)

‘… the area that gives me anxiety is the fact that I haven’t been able to do my core function along with it, because every time there’s an emergency, especially within the COVID area, I have to drop my core function and go and run to sort that out.’ (P5, Male, Physiotherapist)

‘Because everybody was afraid, and everybody was so unsure of what was happening. It made life very difficult.’ (P12, Female, Manager)

‘So that (Not being home) also created another level of being anxious and calling and finding out. How are you doing? Do you need anything? Trying to find a long-lost friend back home, who can go do something quickly, and drop it off for your mother.’ (P12, Female, Manager)

The notion of rehabilitation practitioners not displaying their vulnerability was quickly discarded as many created healing and holding spaces for their colleagues. Rehabilitation practitioners shared their own COVID-19 stories; the aims for this were multifold. Some saw it as a means to break the stigma of being COVID-19 positive, especially during the early stages when even health care workers were unsure how to respond. Storytelling was used to establish a broader sense of belonging and healing. This ‘unmasking of emotions’ represents a significant shift in how rehabilitation practitioners overtly engage their own humanity in relation to their personal health risks, the Loss or death of family and loved ones. Practices, mediated by anxiety, fear, self-advocacy and suchlike created a humanising professional development experience and highlighted the spirit of ubuntu. The following responses from participants display how ubuntu was ignited within healthcare settings. Rehabilitation practitioners also reached out to one another and offered support; many online platforms and resources were also shared.

‘If I can say it humanity, we got to learn about each other as humans, and what makes us human and what makes us tick and what makes us scared.’ (P12, Female, Manager)

‘The physio manager would come to me and say: “I am so tired of seeing patients dying. We can’t anymore, we really can’t.” And so in those conversations it would come up, okay tell me how my staff can help you? How can we help you? What can we do to ease the burden on you guys?’ (P3, Female, Manager)

### ‘Think out of the box … think on our feet’

The importance of putting patients’ rights first (Uys et al. 2021) was emphasised by participants and in some places, patients were provided with varied options in terms of service preference and attendance. Rehabilitation practitioners had to find new ways of practice. Telerehabilitation is an effective method to reduce the financial burden and improve access to healthcare services for patients and their families in low socio-economic settings (WHO 2021). In this study, rehabilitation practitioners reported that the initial lockdown period provided them with the opportunity to investigate telerehabilitation practices and how it could improve access to healthcare services:

‘… we had to be resourceful; we had to be really innovative.’ (P3)

‘… we all had to think out of the box, we all had to think on our feet.’ (P12, Female, Manager)

The telerehabilitation practices utilised included both synchronous (video calls) and asynchronous (pre-recorded videos and WhatsApp groups) methods. Rehabilitation practitioners reported that they effectively reached treatment aims through the distribution of pre-recorded videos which assisted patients with activities like dressing and completing exercises within their home environment. Pre-recorded videos were also used to train caregivers and family members of patients who were unable to enter health facilities for essential practical training such as wheelchair transfers and home exercises. WhatsApp groups were created to maintain communication and support as another asynchronous method of service delivery.

The socio-economic status of rehabilitation practitioners determined whether they were able to personally access the technology required. Some were able to use their own technology like cellular phones, tablets and their own data to provide telerehabilitation, as resources were unavailable at institutions. Rehabilitation practitioners at one site approached cellular network providers for assistance and funding to ensure telerehabilitation services were available for themselves and for their patients. The need for equitable access to resources for telehealth was highlighted by Participant 6:

‘… our telehealth was not that advanced one as in you’re going to Skype with the patient or Zoom the patient, we were mostly checking them telephonically to find out, how are they doing.’ (P6, Male, Audiologist)

To date, there are no published guidelines on telerehabilitation by the National Department of Health to guide practitioners and managers (Govender et al. 2022). The need for these guidelines were expressed by Participant 1:

‘we need to develop proper, clear guidelines on how we implement telerehabilitation or telemedicine so that the services we provide are not diluted.’ (P1, Male, Manager)

### An epilogue on race

In the context of South Africa’s history, race and racism have deeply influenced all parts of society, including healthcare and rehabilitation workers. While the discussions with participants didn’t always directly highlight the role of race in rehabilitation, the conversations did emphasise the significance of race-related issues in South Africa, such as economic disparities and unequal healthcare services.

However, participants’ understanding of their social and professional identities, including factors like race and gender, had an indirect influence on how they saw their role as practitioners. For example, Participant 2 mentioned that therapists from different backgrounds might have varying levels of exposure to different environments, impacting their ability to relate to patients’ challenges, like lack of access to healthcare because of financial constraints and transportation issues.

Interestingly, one participant noted that factors like race, gender and age weren’t as crucial as the amount of clinical experience they had. This viewpoint contrasts with a previous argument by Pillay and Pillay (2021) that highlighted the role of race and social identities in rehabilitation practice, particularly in cases like dysphagia rehabilitation in South Africa.

Nonetheless, further investigation is needed to fully establish the significance of race and racism as key factors in how practitioners responded during the pandemic.

### Study critique

This study presents a cross-professional examination of rehabilitation practices during the pandemic, highlighting the growing relevance beyond traditional healthcare domains such as medical and nursing services. By doing so, it sheds light on the distinct contextual challenges faced by rehabilitation practitioners in South Africa. It should be noted that this study provides a comprehensive portrayal of rehabilitation practitioners, predominantly from therapeutic disciplines, while excluding input from stakeholders in fields like psychology, social work and other healthcare professions. This study’s scope does not encompass a specific geographical or socio-economic context. However, it offers valuable insights as a representative voice from the public rehabilitation service sector, which has been the primary resource for many South Africans with disabilities during the pandemic. The experiences recounted by South African rehabilitation practitioners in this study could potentially resonate with those of professionals in similar settings across the globe, particularly in low-to-middle-income countries.

Methodologically, the inclusion of a non-rehabilitation partner, in this case a journalist, for critical discussions is noteworthy. This study provides, via the use of critical conversations, a robust approach to studying the experiences of rehabilitation practitioners. When synthesising the interview data, it was noted that there was potential for uncovering valuable insights into the diverse and challenging landscape of rehabilitation practice in South Africa. As a method ‘critical conversation’ may benefit from more explicit review of how, more specifically its techniques and methods may be used to foreground social, economic and political issues associated with rehabilitation practice in South Africa. Its integration within a phenomenological approach allowed for nuanced revelations on social and cultural influences on rehabilitation practitioners. This innovative approach not only introduces external perspectives but also brings essential critical perspectives to the forefront. Moreover, it holds the promise of generating fresh insights into the societal, cultural and political dimensions of rehabilitation practice.

## Conclusion

This study explored the lived experiences of rehabilitation practitioners in South Africa during the COVID-19 pandemic, shedding light on the profound shifts and challenges they encountered. Through an in-depth exploration of their narratives, three significant themes emerged: ‘Management became the enemy’, ‘Tired of being resilient’, and ‘Think out of the box … think on our feet’.

In summary, the COVID-19 pandemic catalysed shifts in leadership dynamics, emotional responses and service delivery practices among rehabilitation practitioners in South Africa. The study reveals a multidimensional narrative that encompasses resilience, vulnerability, innovation and the intricacies of social identity. This also affirms the voices of rehabilitation practitioners in a public platform. The findings on rehabilitation professions in South Africa hold the potential to significantly enhance future pandemic planning and preparedness. By analysing the experiences, challenges and adaptations of rehabilitation professionals during and after the pandemic, this study can provide valuable insights into the resilience of health care systems, the effectiveness of remote and virtual rehabilitation services, the impact of patient outcomes, and the identification of critical gaps in the healthcare workforce. The findings may serve as a foundational resource for policy makers, healthcare administrators and public health experts, enabling them to devise more robust strategies for integrating rehabilitation services into pandemic response frameworks, optimising resource allocation, and ensuring the continuity of essential healthcare services even under challenging circumstances.

As the healthcare landscape continues to evolve, the insights garnered provide a platform for understanding, reflection, and reform within the realm of rehabilitation practice. It calls for an ongoing commitment to address the challenges identified, cultivate inclusive, collective leadership, prioritise practitioner well-being and harness the potential of innovative solutions to enhance healthcare delivery and outcomes.

## References

[CIT0001] Abrahams, K., Kathard, H., Harty, M. & Pillay, M., 2019, ‘Inequity and the professionalisation of speech-language pathology’, *Professions and Professionalism* 9(3). 10.7577/pp.3285

[CIT0002] Adams, S.N., Seedat, J., Coutts, K. & Kater, K-A., 2021, ‘We are in this together’ voices of speech-language pathologists working in South African healthcare contexts during level 4 and level 5 lockdown of COVID-19’, *South African Journal of Communication Disorders* 68(1), a792. 10.4102/sajcd.v68i1.792 PMC8007987 33764150

[CIT0003] Ahmed, U.M., Hanif, M., Ali, M.J., Haider, M.A., Kherani, D., Memon, G.M. et al., 2020, ‘Neurological manifestations of COVID-19 (SARS-COV-2): A review’, *Frontiers in Neurology* 11, 518. 10.3389/fneur.2020.00518 32574248 PMC7257377

[CIT0004] Aliyu, S., Travers, J.L., Norful, A.A., Clarke, M. & Schroeder, K., 2021, ‘The lived experience of being diagnosed with COVID-19 among Black patients: A qualitative study’, *Journal of Patient Experience* 8, 1–9. 10.1177/2374373521996963 PMC8205343 34179380

[CIT0005] Amatya, B. & Khan, F., 2020a, ‘COVID-19 in developing countries: A rehabilitation perspective’, *The Journal of the International Society of Physical and Rehabilitation Medicine* 3(2), 69–74. 10.4103/jisprm.jisprm_12_20

[CIT0006] Amatya, B. & Khan, F., 2020b, ‘Rehabilitation response in pandemics’, *American Journal of Physical Medicine & Rehabilitation* 99(8), 663–668. 10.1097/PHM.0000000000001477 32452879 PMC7268860

[CIT0007] Anjara, S., Fox, R., Rogers, L., De Brún, A. & McAuliffe, E., 2021, ‘Teamworking in healthcare during the COVID-19 pandemic: A mixed-method study’, *International Journal of Environmental Research and Public Health* 18, 10371. 10.3390/ijerph181910371 34639671 PMC8508523

[CIT0008] Archibald, M.M., Ambagtsheer, R.C., Casey, M.G. & Lawless, M., 2019, ‘Using zoom videoconferencing for qualitative data collection: Perceptions and experiences of researchers and participants’, *International Journal of Qualitative Methods* 18, 1–8. 10.1177/1609406919874596

[CIT0009] Banks, L.M., Davey, C., Shakespeare, T. & Kuper, T., 2020, ‘Disability-inclusive responses to COVID-19: Lessons learnt from research and social protection in low- and middle income countries’, *World Development* 137, 105178. 10.1016/j.worlddev.2020.105178 32904300 PMC7455235

[CIT0010] Braun, V. & Clarke, V., 2019, ‘Reflecting on reflexive thematic analysis’, *Qualitative Research in Sport, Exercise & Health* 11(4), 589–597. 10.1080/2159676X.2019.1628806

[CIT0011] Cag, Y., Edeem, H., Gormez, A., Ankarali, H., Hargreaves, S., Ferreira-Coimbra, J. et al., 2021, ‘Anxiety among front-line health care workers supporting patients with Covid 19: A global survey’, *General Hospital Psychiatry* 68, 90–96. 10.1016/j.genhosppsych.2020.12.010 33418193 PMC7749993

[CIT0012] Chersich, M.F., Gray, G., Fairlie, L., Eichbaum, Q., Mayhew, S., Allwood, B. et al., 2020, ‘COVID-19 in Africa: Care and protection for frontline healthcare workers’, *Global Health* 16(1), 46. 10.1186/s12992-020-00574-3 32414379 PMC7227172

[CIT0013] Dawood, B., Tomita, A. & Ramlall, S., 2022, ‘“Unheard”, “uncared for” and “unsupported”: The mental health impact of Covid-19 on healthcare workers in KwaZulu-Natal Province, South Africa’, *PLoS ONE* 17(5), e0266008. 10.1371/journal.pone.0266008 35507540 PMC9067674

[CIT0014] De Biase, S., Cook, L., Skelton, D.A., Witham, M. & Ten Hove, R., 2020, ‘The COVID-19 rehabilitation pandemic’, *Age and Ageing* 49(5), 696–700. 10.1093/ageing/afaa118 32470131 PMC7314277

[CIT0015] Delve, H.L. & Limpaecher, A., 2022, ‘The importance of reflexivity in qualitative research’, in *Essential guide to coding qualitative data*, viewed 07 January 2022, from https://delvetool.com/blog/reflexivity.

[CIT0016] De Villiers, K., 2021, ‘Bridging the health inequality gap: An examination of South Africa’s social innovation in health landscape’, *Infectious Diseases of Poverty* 10, 19. 10.1186/s40249-021-00804-9 33648585 PMC7919075

[CIT0017] Dierckx de Casterle, B., Verhaeghe, S., Kars, M.C., Coolbrandt, A., Stevens, M., Stubbe, M. et al., 2011, ‘Researching lived experience in health care: Significance for care ethics’, *Nursing Ethics* 18(2), 232–242. 10.1177/0969733010389253 21372236

[CIT0018] Doraiswamy, S., Abraham, A., Mamtani, R. & Cheema, S., 2020, ‘Use of telehealth during the COVID-19 pandemic: Scoping review’, *Journal of Medical Internet Research* 22(12), e24087. 10.2196/24087 33147166 PMC7710390

[CIT0019] Edwards, G. & Bolden, R., 2023, ‘Why is collective leadership so elusive?’, *Leadership* 19(2), 167–182. 10.1177/17427150221128357

[CIT0020] Fathi, E., Malekshahi-Beiranvand, F., Nobahari, A., Daneshpour, M. & hatami-Vazaneh, A., 2020, ‘The lived experience of health care workers during the coronavirus outbreak: A content analysis study’, *International Journal of Behavioural Sciences* 14(3), 161–166. 10.30491/ijbs.2020.248645.1368

[CIT0021] Feijit, M., De Kort, Y., Bongers, I., Bierbooms, J., Westerink, J. & IJsselsteijn, W., 2020, ‘Mental health care goes online: Practitioners’ experiences of providing mental health care during the COVID 19 pandemic’, *Cyberpsychology, Behaviour and Social Networking* 23(12), 860–864. 10.1089/cyber.2020.0370 32815742

[CIT0022] Finlay, F., 2009, ‘Exploring lived experience: Principles and practice of phenomenological research’, *International Journal of Therapy and Rehabilitation* 16(9), 474–481. 10.12968/ijtr.2009.16.9.43765

[CIT0023] Firshman, P., Hoffman, K. & Rapolthy-Beck, A., 2020, *Occupational therapy for COVID-19 patients in ICU and beyond*, Intensive Care Society, viewed 04 December 2021, from https://ics.ac.uk/resource/occupational-therapy-for-covid-19-patients-in-icu.html.

[CIT0024] Francisco, M., Olmos-Vega, Renée, E., Stalmeijer, Lara Varpio & Renate Kahlke, 2023, ‘A practical guide to reflexivity in qualitative research: AMEE Guide No. 149’, *Medical Teacher* 45(3), 241–251. 10.1080/0142159X.2022.2057287 35389310

[CIT0025] Frechette, J., Bitzas, V., Aubry, M., Kilapatrick, K. & Lavoie-Tremblay, M., 2020, ‘Capturing lived experience: Methodological considerations for interpretive phenomenological inquiry’, *International Journal of Qualitative Methods* 19, 1–12. 10.1177/1609406920907254

[CIT0026] Gilgun, J. & Jane, F., 2008, ‘Lived experience, reflexivity, and research on perpetrators of interpersonal violence’, *Qualitative Social Work* 7(2), 181–197. 10.1177/1473325008089629

[CIT0027] Giraldo-Pedroza, A., Robayo-Torres, A.L., Guerrero, A.V.S. & Nicholls, D.A., 2021, ‘Narrative histories of physiotherapy in Colombia, Ecuador, and Argentina’, *Physiotherapy Theory & Practice* 37(3), 447–459. 10.1080/09593985.2021.1887059 33678111

[CIT0028] Given, L.M., 2008, *The SAGE encyclopedia of qualitative research methods*, vols. 1–0, SAGE, Thousand Oaks, CA.

[CIT0029] Govender, S., Vallabhjee, A.L., Charles, C.R., Roesch, D. & Balton, S., 2022, ‘Bridging the access gap: The telepractice experience of speech therapists and audiologists at a public health care facility in South Africa’, *International Journal of Telerehabilitation* 14(2), e6517–e6517. 10.5195/ijt.2022.6517 38026559 PMC10681049

[CIT0030] Hassem, T., Israel, N., Bemath, N. & Variava, T., 2022, ‘COVID-19: Contrasting Experiences of South African Physiotherapists Based on Patient Exposure’, *The South African Journal of Physiotherapy* 78(1), 1576–1576. 10.4102/sajp.v78i1.1576 35169652 PMC8831904

[CIT0031] Hagens, V., Dobrow, M.J. & Chafe, R., 2009, ‘Interviewee transcript review: Assessing the impact on qualitative research’, *BMC Medical Research Methodology* 9, 1–8. 10.1186/1471-2288-9-47 19580666 PMC2713273

[CIT0032] Hill, R., Butnoris, M., Dowling, H., Macolino, K., Patel, B., Simpson, T. et al., 2020, ‘Reflection on our leadership during COVID-19: Challenging our resilience’, *Journal of Pharmacy Practice and Research* 50, 294–296. 10.1002/jppr.1681

[CIT0033] Honey, A., Boydell, K.M., Caniglio, F., Trang, T.D., Gill, K., Glover, H. et al., 2020, ‘Lived experience research as a resource for recovery: A mixed methods study’, *BMC Psychiatry* 10(456), 1–13. 10.1186/s12888-020-02861-0 PMC7507671 32958045

[CIT0034] Iheduru-Anderson, K., 2020, ‘Reflections on the lived experience of working with limited personal protective equipment during the COVID 19 crises’, *Nursing Inquiry* 28(1), e12382. 10.1111/nin.12382 33010197 PMC7646033

[CIT0035] Ilyas, A., Naiz, A., Abualait, T. & Bashir, S., 2021, ‘The impact of COVID-19 pandemic on rehabilitation services in a tertiary care hospital in the Eastern Region of Saudi Arabia: A single-center study’, *Cureus* 13(9), e18303. 10.7759/cureus.18303 34722078 PMC8548602

[CIT0036] Jow, S., Doshi, S., Desale, S. & Malmut, L., 2022, ‘Mental health impact of COVID-19 pandemic on therapists at inpatient rehabilitation facility’, *The Journal of Injury, Function and Rehabilitation* 15(2), 168–175. 10.1002/pmrj.12860 PMC9347797 35666036

[CIT0037] Kamal, M., Abo Omirah, M., Hussein, A. & Saeed, H., 2021, ‘Assessment and characterisation of post COVID-19 manifestations’, *International Journal of Clinical Practice* 75(3), e13746. 10.1111/ijcp.13746 32991035 PMC7536922

[CIT0038] Karimi, Z., Fereidouni, Z., Behnammoghadam, M., Alimohammadi, N., Mousavizaden, A. & Mirzaee, S., 2020, ‘The lived experience of nurses caring for patients with COVID-19 in Iran: A phenomenological study’, *Risk Management and Health Care Policy* 13, 1271–1278. 10.2147/RMHP.S258785 PMC7450521 32904130

[CIT0039] Lambert, V., Glacken, M. & McCarron, M., 2011, ‘Employing an ethnographic approach: Key characteristics’, *Nurse Researcher* 19(1), 17–23. 10.7748/nr2011.10.19.1.17.c8767 22128583

[CIT0040] Landes, S.D., Turk, M., Ashlyn, A.W. & Wong, P.A., 2020, ‘COVID-19 outcomes among people with intellectual and developmental disabilities in California: The importance of type of residence and skilled nursing care needs’, *Disability and Health Journal* 14. https://doi.org.10/1016/j.dhjo.2020.101051 10.1016/j.dhjo.2020.101051 PMC7719000 33309535

[CIT0041] Lebrasseur, A., Fortin-Bédard, N., Lettre, J., Bussières, E.-L., Best, K., Boucher, N., et al., 2021, ‘Impact of COVID-19 on people with physical disabilities: A rapid review’, *Disability and Health Journal* 14(1), 101014–101014. 10.1016/j.dhjo.2020.101014 33158795 PMC7603994

[CIT0042] Lee, H.L., Wilson, K.S., Bernstein, C., Naicker, N., Yassi, A. & Spiegel, J.M., 2022, ‘Psychological distress in South African healthcare workers early in the COVID-19 pandemic: An analysis of associations and mitigating factors’, *International Journal of Environmental Research and Public Health* 19(15), 9722. 10.3390/ijerph1915972 35955078 PMC9368661

[CIT0043] Leochico, C.F.D., Rey-Matias, B.M.V. & Rey-Matias, R.R., 2022, ‘Telerehabilitation perceptions and experiences of physiatrists in a lower-middle-income country during the COVID-19 pandemic’, *PM &R* 14(2), 210–216. 10.1002/pmrj.12715 34585855 PMC8661588

[CIT0044] Lew, H.L., Oh-Park, M. & Cifu, D.X., 2020, ‘The war on COVID-19 pandemic: Role of rehabilitation professionals and hospitals’, *American Journal of Physical Medicine and Rehabilitation* 99(7), 571–572. 10.1097/PHM.0000000000001460 32371624 PMC7268823

[CIT0045] Liu, Q., Luo, D., Haase, J., Guo, Q., Wang, X.Q., Liu, S. et al., 2020, ‘The experiences of health-care providers during the COVID-19 crises in China: A qualitative study’, *Lancet Global Health* 8(6), e790–799. 10.1016/S2214-109X(20)30204-7 32573443 PMC7190296

[CIT0046] Lund, E., Forber-Pratt, A., Wilson, C. & Mona, L., 2020, ‘The COVID-19 pandemic, stress, and trauma in the disability community: A call to action’, *Rehabilitation Psychology* 65(4), 313–322. 10.1037/rep0000368 33119381

[CIT0047] Macnamara, J., 2021, ‘New insights into crisis communication from an “inside” emic perspective during COVID-19’, *Public Relations Inquiry* 10(2), 237–262. 10.1177/2046147X21999972

[CIT0048] Malec, J., Salisbury, D., Anders, D., Dennis, L., Groff, A., Johnson, M. et al., 2020, ‘Response to the COVID-19 pandemic among posthospital brain injury rehabilitation providers’, *Archives of Physical Medicine and Rehabilitation* 102(3), 549–555. 10.1016/j.apmr.2020.10.137 33253694 PMC7695439

[CIT0049] Mather, P., 2020, ‘Leadership and governance in a crisis: Some reflections on COVID-19’, *Journal of Accounting & Organizational Change* 16(4), 579–585. 10.1108/JAOC-08-2020-0123

[CIT0050] McKinney, E.L., McKinney, V. & Swartz, L., 2021, ‘Access to healthcare for people with disabilities in South Africa: Bad at any time, worse during COVID-19?’, *South African Family Practice: Official Journal of the South African Academy of Family Practice/Primary Care* 63(1), e1–e5. 10.4102/safp.v63i1.5226 PMC8335793 34342484

[CIT0051] Modisenyane, M., Mngemane, S., Maolela, T., Nemungadi, T., Chituku, P., Leonard, E. et al., 2021, ‘Test-trace strategy for disease control and management: South Africa’s control measures to contain the spread of COVID-19’, in K. Govender, G. George, A. Padarath & T. Moeti (eds.), *South African health review 2021 health sector responses to COVID-19*, pp. vii–ix, Health Systems Trust, viewed 20 February 2022, from https://www.hst.org.za/publications/Pages/SAHR2021.

[CIT0052] Ned, L., McKinney, E.L.M., McKinney, V. & Swartz, L., 2020, ‘COVID-19 pandemic and disability: Essential considerations’, *Social and Health Sciences* 18(2), 136–148.

[CIT0053] Ngeh, N.E., Chigbo, N.N., Whitehouse, Z., Anekwu, E.M., Mukaruzima, L., Mtsetfwa, L. et al., 2021, ‘A report on the development of COVID-19 guidelines for rehabilitation professionals in African settings’, *The Pan African Medical Journal* 38(129), 129–129. 10.11604/pamj.2021.38.129.26311 33912299 PMC8051208

[CIT0054] Nkonki, L. & Fonn, S., 2020, ‘Decisive and strong leadership and intersectoral action from South Africa in response to the COVID-19 virus’, *SAMJ South African Medical Journal* 110(5), 339–340. 10.7196/SAMJ.2020.v110i5.14739 32657709

[CIT0055] Olive, J.L., 2014, ‘Reflecting on the tensions between emic and etic perspectives in life history research: Lessons learned’, *Forum, Qualitative Social Research* 15(2).

[CIT0056] Olmos-Vega, F.M., Stalmeijer, R.E., Varpio, L. & Kahlke, R., 2023, ‘A practical guide to reflexivity in qualitative research: AMEE Guide No. 149’, *Medical Teacher* 45(3), 241–251. 10.1080/0142159X.2022.2057287 35389310

[CIT0057] Patterson, J., Govender, R., Clunie, G., Murphy, J., Brady, G., Haines, J. et al., 2020, ‘COVID-19 and ENT speech, language therapy services workforce and research in the UK: A discussion paper’, *International Journal of Communication Disorders* 55(5), 806–817. 10.1111/1460-6984.12565 PMC7436215 32770652

[CIT0058] Pillay, M., 2003, ‘(Re)positioning the powerful expert and the sick person: The case of communication pathology’, Unpublished doctoral dissertation, University of Durban-Westville.

[CIT0059] Pillay, T. & Pillay, M., 2021, ‘The power struggle: Exploring the reality of clinical reasoning’, *Health* 27(4), 559–587. 10.1177/13634593211054008 34763576

[CIT0060] Pillay, M., Tiwari, R., Kathard, H. & Chikte, U., 2020, ‘Sustainable workforce: South African audiologists and speech therapists’, Human Resources for Health 18(1), 1–13. 10.1186/s12960-020-00488-6 32611357 PMC7329495

[CIT0061] Pinilla-Roncancio, M., 2015, ‘Disability and poverty: Two related conditions. A review of the literature’, *Revista de la Facultad de Medicina* 63(suppl. 1), 113–123. 10.15446/revfacmed.v63n3sup.50132

[CIT0062] Portacolone E., Chodos A., Halpern J., Covinsky K.E., Keiser S., Fung J., et al., 2021, ‘The Effects of the COVID-19 Pandemic on the Lived Experience of Diverse Older Adults Living Alone With Cognitive Impairment’, *The Gerontologist* 61(2), 251–261. 10.1093/geront/gnaa201. 33404634 PMC7901518

[CIT0063] Ramose, M.B., 2002, ‘The philosophy of ubuntu and ubuntu as a philosophy’, in P.H. Coetzee & A.P.J. Roux (eds.), *Philosophy from Africa: a text with readings*, pp. 230–237, Oxford University Press, Oxford.

[CIT0064] Roberts, E.M., Knestrick, J. & Resick, L., 2021, ‘The lived experience of COVID 19’, *The Journal for Nurse Practitioners* 17(7), 828–832. 10.1016/j.nurpra.2021.04.013 33935606 PMC8075807

[CIT0065] Robertson, L.J., Maposa, I., Samaroo, H. & Johnson, O., 2020, ‘Mental health of healthcare workers during COVID 19 outbreak: A rapid scoping review to inform provincial guidelines in South Africa’, *South African Medical Journal* 11(10), 1010–1019. 10.7196/SAMJ.2020.v110i10.15022 33205731

[CIT0066] Royal College for Speech, Language Therapy (RCSLT), 2022, *Understanding the need for and provision of speech and language services for individuals with post-covid syndrome in the UK*, viewed 20 December 2022, from https://www.rcslt.org/news/long-covid-and-sustained-impact-reports/.

[CIT0067] Saragih, I.D., Tonapa, S.I., Saragih, I.S., Advani, S., Batubara, S.O., Suarilah, I. et al., 2021, ‘Global prevalence of mental health problems among healthcare workers during the COVID-19 pandemic: A systematic review and meta-analysis’, *International Journal of Nursing Studies* 121, 104002. 10.1016/j.ijnurstu.2021.104002 34271460 PMC9701545

[CIT0068] Sheehy, L.M., 2020, ‘Considerations for postacute rehabilitation for survivors of COVID-19’, *JMIR Public Health Surveillance* 6(2), 19462. 10.2196/19462 PMC7212817 32369030

[CIT0069] Sveningsson, S. & Kärreman, D., 2021, ‘Alvesson, mats: A passion for critical reflexivity and rational change’, in D.B. Szabla (ed.), *The Palgrave handbook of organizational change thinkers*, pp. 17–35, Palgrave Macmillan, Cham.

[CIT0070] Temsah, M.H., Al-Sohime, F., Alamro, N., Al-Eyadhy, A., Al-Hasan, K., Jamal, A. et al., 2020, ‘The psychological impact of COVID 19 pandemic on health care workers in a MERS-CoV endemic country’, *Journal of Infection and Public Health* 13, 877–882. 10.1016/j.jiph.2020.05.021 32505461 PMC7256548

[CIT0071] Uys, K., Casteleijn, D., Van Niekerk, K., Balbadur, R., d’Oliveira, J. & Msimango, H., 2021, ‘The impact of COVID-19 on occupational therapy services in Gauteng Province, South Africa: A qualitative study’, in K. Govender, G. George, A. Padarath & T. Moeti (eds.), *South African health review: Health sector responses to COVID-19*, pp. vii–ix, Health Systems Trust, viewed 02 February 2022, from https://www.hst.org.za/publications/Pages/SAHR2021.

[CIT0072] World Health Organization (WHO), 2020, *Coronavirus disease 2019 (COVID 19): Situation report*, 82, viewed 26 November 2021, from https://apps.who.int/iris/handle/10665/331780.

[CIT0073] World Health Organization (WHO), 2021, *Leveraging telehealth for efficient delivery of primary health care in the WHO South East Asia Region*, viewed 02 January 2022, from https://apps.who.int/iris/handle/10665/350199.

[CIT0074] Yarrow, E. & Pagan, V., 2021, ‘Reflections on front-line medical work during COVID-19 and the embodiment of risk’, *Feminist Frontiers* 28(51), 89–100. 10.1111/gwao.12505 PMC7361443 32837018

